# Nonlinear association of cholesterol, high-density lipoprotein, and glucose index with the risk of prediabetes and diabetes in normal-weight middle-aged and older Chinese adults: a multicenter retrospective cohort study

**DOI:** 10.3389/fnut.2026.1949954

**Published:** 2026-10-19

**Authors:** Junling Ding, Zhimao Cai, Xianglin Wang, Lifen Qin, Weifeng Chen

**Affiliations:** 1 Dapeng New District Kuichong Branch of Shenzhen Second People’s Hospital, Shenzhen, Guangdong, China; 2 Sanxigaoyuan Community Health Service Center of ShenZhen Dapeng New District Medical and Health Group, Shenzhen, Guangdong, China; 3 Department of General Medicine, Shenzhen Second People’s Hospital, The First Affiliated Hospital of Shenzhen University, Shenzhen, Guangdong, China; 4 Department of General Practice, Shenzhen University Health Science Center, Shenzhen, Guangdong, China; 5 Department of Internal Medicine, Dapeng New District Kuichong Branch of Shenzhen Second People’s Hospital, Shenzhen, Guangdong, China; 6 Shenzhen Dapeng New District Medical and Health Group, Shenzhen, Guangdong, China

**Keywords:** cohort study, diabetes, middle-aged and older, nonlinear association, normal-weight, prediabetes

## Abstract

**Background:**

The relationship between the cholesterol, high-density lipoprotein, and glucose (CHG) index and dysglycemia is widely recognized in the general population; however, this relationship has been much less examined among normal-weight, middle-aged and older adults—a subgroup routinely marginalized in metabolic research. Accordingly, the current study was designed to evaluate the relationship of the CHG index with incident prediabetes and diabetes specifically among this population.

**Methods:**

The study cohort comprised 19,981 Chinese adults (age ≥45 years, BMI 18.5–23.9 kg/m^2^) without prediabetes or diabetes at baseline, sourced from a multicenter retrospective analysis of nationwide screening data (2010–2016). The CHG index was calculated as Ln[(TC × FPG)/(2 × HDLC)] (mg/dL). The primary outcome was incident- prediabetes and diabetes. To evaluate the association, we applied Cox proportional hazards models, along with smooth curve fitting and two-piecewise regression.

**Results:**

During an average follow-up of 3.19 years, a higher CHG index was significantly linked to a greater risk of the outcome. After adjustment (Model 3), the hazard ratio (HR) for the CHG index as a continuous variable was 2.20 (95% CI: 1.90–2.54). Compared with Quartile 1, the HRs for Quartiles 2, 3, and 4 were 1.42, 1.86, and 1.91, respectively (all *p* < 0.0001). A significant nonlinear relationship was identified (log-likelihood ratio test, *p* < 0.001), with the inflection point located at a CHG index of 5.07. To the left of the inflection point, each unit increase in CHG index was associated with a 6.39-fold increase in risk (HR: 6.39, 95% CI: 4.73–8.64), while no significant link was observed to the right (HR: 1.02, 95% CI: 0.79–1.31).

**Conclusion:**

This study indicates a nonlinear positive association between the CHG index and the risk of incident prediabetes and diabetes among normal-weight middle-aged and older Chinese adults. Future research employing large prospective cohorts is warranted to fully explicate the mechanisms and establish a causal nexus.

## Introduction

Diabetes mellitus (DM), a chronic metabolic disease featuring persistent hyperglycemia, has increased continuously in global prevalence over recent decades, positioning itself as one of the major non-communicable diseases threatening public health (1, 2). The pathological underpinnings of this condition involve insufficient insulin secretion or diminished insulin sensitivity in target organs, which can precipitate microvascular and macrovascular damage, markedly elevating the probability of long-term complications such as cardiovascular events, diabetic nephropathy, and retinopathy (3, 4). The direct and indirect medical outlays have posed a major fiscal challenge to healthcare systems worldwide (5). The latest epidemiological figures from the International Diabetes Federation indicate that, in 2021, the number of individuals aged 20–79 years living with DM globally stood at 537 million, with China accounting for approximately 141 million cases—the highest worldwide—and this number is projected to rise further by 2045 (6). In contrast to DM, prediabetes represents a more insidious state of glucoregulatory decompensation, manifested by fasting or postprandial glucose levels exceeding the normal upper limit without yet crossing the diagnostic threshold for DM (7). Owing to the general absence of overt clinical manifestations at this stage, vast numbers of individuals with prediabetes remain unaware of their metabolic abnormalities; epidemiological investigations indicate that over 300 million adults globally are affected, with an estimated 5–10% of patients with prediabetes irreversibly progressing to clinical DM each year (8, 9). Of particular importance is the observation that among middle-aged and older adults whose body mass index falls within the normal reference interval, DM and prediabetes often lack the typical obese phenotype that might serve as a warning sign (10). Although outwardly lean, these individuals may have intrinsic metabolic defects such as ectopic visceral fat accumulation and peripheral insulin resistance—a phenotype termed “metabolically obese normal weight” (10). Existing clinical evidence demonstrates that the atherosclerotic and adverse cardiovascular event risks in such individuals are comparable to that in those with overt obesity; nevertheless, because conventional risk scoring models rely excessively on anthropometric indices such as BMI, this substantial population has remained chronically underidentified and unaddressed (11, 12).

In recent years, the cholesterol, high-density lipoprotein, and glucose (CHG) index has attracted attention as a novel composite metabolic indicator. This index is constructed by combining three routine blood biomarkers, consisting of total cholesterol (TC), high-density lipoprotein cholesterol (HDL-C), and fasting plasma glucose (FPG), which collectively yield concurrent data on lipid burden, reverse cholesterol transport capacity, and glycemic status (13). The CHG index has been reported to be significantly correlated with DM risk in earlier studies (14). Furthermore, cross-sectional evidence has confirmed a nonlinear link between the CHG index and DM prevalence (15). Beyond diabetes, CHG index has demonstrated predictive value for diabetic microvascular complications and cardiovascular endpoints, with higher CHG index linked to diabetic retinopathy and nephropathy in diabetes and to broader cardiovascular risk signals including hypertension, stroke, and mortality in aggregate analyses (16–18). The CHG index may serve as a cost-effective and convenient instrument for the early detection of high-risk individuals, given its straightforward derivation from routine lipid and glucose measurements at low cost.

Notably, despite the CHG index having been demonstrated to be significantly linked to dysglycemia risk in general and prediabetic populations, its role in normal-weight middle-aged and older adults is unknown. Normal weight does not guarantee metabolic health; many such individuals have abdominal obesity and insulin resistance—the “metabolically unhealthy normal weight” phenotype—and elevated diabetes risk (19, 20). Among Chinese middle aged and older populations, even among those with normal weight, metabolic abnormalities may exert a greater influence on diabetes development than obesity per se (19, 20). Therefore, investigating the link between the CHG index and dysglycemia risk in this seemingly “low risk” yet metabolically vulnerable population carries important clinical and public health implications (19–21). Using data from a multicenter retrospective cohort, this study aims to systematically evaluate the link between the CHG index and the risk of prediabetes and diabetes among normal-weight middle-aged and older Chinese adults, with a particular focus on exploring potential nonlinear relationships and threshold effects, thereby contributing to evidence-based risk classification and tailored prevention strategies for this group.

## Methods

### Study design and population

This retrospective study performed a secondary analysis of a publicly available dataset that contained 211,833 Chinese adults, which was initially established by Chen et al. (22) and has been accessible via the Dryad Digital Repository.^1^ This repository operates as an open-access platform, authorizing the downloading and reuse of its contents for scientific purposes under its established policies. Ethical oversight for the primary data collection was provided by the Rich Healthcare Group Review Board; consequently, the current secondary investigation did not necessitate separate ethical clearance. All procedures performed in both the antecedent and current studies were congruent with the ethical guidelines delineated in the Declaration of Helsinki.

Between 2010 and 2016, a total of 685,277 Chinese adults (aged ≥20 years) were enrolled in health examination programs conducted at 32 screening sites across 11 cities in China. Following the sequential exclusion of cases with missing data, physiologically implausible measurements, preexisting diabetes at baseline, and an insufficient follow-up period, the final analytical cohort was reduced to 211,833 individuals.

To further refine the analytical sample for the current work, which exclusively enrolled normal-weight, middle-aged and elderly participants, additional eligibility criteria were applied. Participants were sequentially excluded if they met any of the following criteria: (1) age <45 years (*n* = 136,462); (2) body mass index (BMI) outside the normal-weight range, defined as BMI ≥ 24 kg/m^2^ (*n* = 37,714) or BMI < 18.5 kg/m^2^ (*n* = 1,458); (3) missing total cholesterol measurements (*n* = 451); (4) missing high-density lipoprotein cholesterol (HDL-C) measurements (*n* = 10,106); (5) biologically implausible HDL-C values of 0 mmol/L (*n* = 10); (6) baseline fasting plasma glucose (FPG) ≥ 5.6 mmol/L, indicating prevalent prediabetes (*n* = 5,650); and (7) extreme or systematically erroneous CHG index values (<3.5) (*n* = 1). Following these exclusions, we obtained a final analytic cohort of 19,981 participants for the current investigation (Figure 1).

**Figure 1:**
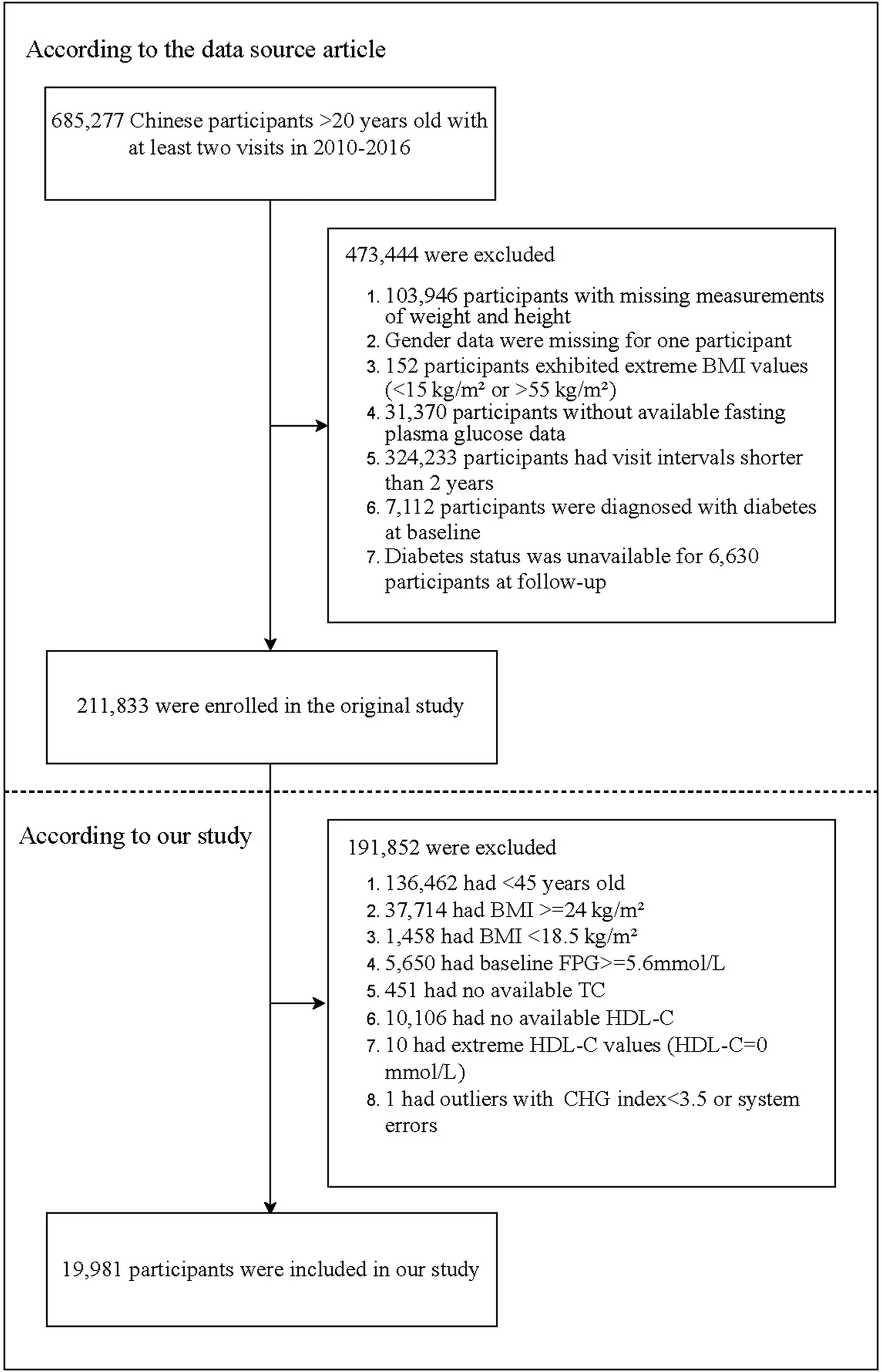
Participants selection flowchart. BMI, body mass index; FPG, fasting plasma glucose; TC, total cholesterol; HDL-C, high-density lipoprotein cholesterol; CHG index, cholesterol, high-density lipoprotein, and glucose index.

### Definitions of the exposure variable

The CHG index, defined as Ln[(TC × FPG)/(2 × HDL-C)], served as the exposure variable (23). Given that the original derivation used mg/dL, we systematically converted FPG (×18) and both TC and HDL-C (×38.67) to mg/dL before substituting them into the formula (24). This step ensured that our calculated indices exactly mirrored the original definition, thereby guaranteeing methodological reproducibility.

### Outcome ascertainment

Prediabetes and diabetes were identified if, during follow-up, participants had a fasting glucose ≥5.6 mmol/L or self-reported diagnosed diabetes at the follow-up examination (25, 26).

### Data collection

In line with previously published methodologies (22), trained personnel conducted all data collection and anthropometric measurements using standardized protocols. Covariates in the present analysis comprised biochemical indices—including alanine aminotransferase (ALT), blood urea nitrogen (BUN), FPG, HDL-C, low-density lipoprotein cholesterol (LDL-C), serum creatinine (Scr), and TC; physical examination data—namely BMI, diastolic blood pressure (DBP), and systolic blood pressure (SBP); demographic characteristics (age and sex); lifestyle factors (drinking and smoking status); and family history of diabetes. Following a minimum 10-h fast, venous blood samples were collected from all participants, and all biochemical assays were carried out on a Beckman 5,800 automated analyzer.

### Statistical analysis

We carried out all statistical analyses with R and EmpowerStats, employing a two-tailed significance threshold of *p* < 0.05. Baseline participant characteristics were summarized descriptively, with continuous data reported as mean ± SD and categorical data as counts and percentages. Survival curves and cumulative incidence rates were derived from the Kaplan–Meier estimator.

Cox proportional hazards regression, from which HRs and their 95% CIs were obtained, was employed to evaluate the relationship of the CHG index with incident prediabetes and diabetes. Three incremental adjustment models were specified: an unadjusted model (Model 1), a model adjusted for DBP, sex, age, and SBP (Model 2), and a model with further adjustment for BMI, LDL-C, Scr, BUN, ALT, and family history of diabetes beyond Model 2 (Model 3). The CHG index was modeled continuously and categorically (quartiles, Q1 as referent), with the P for trend derived from median-based continuous terms.

To examine whether the association between the CHG index and the incidence of prediabetes/diabetes exhibited a nonlinear pattern, we first incorporated smooth curve fitting into the Cox framework for visual inspection. If a nonlinear relationship was suggested, a recursive procedure was then used to locate the turning point, after which a segmented Cox model with two pieces was fitted. Finally, a likelihood ratio test was performed to compare the linear and nonlinear specifications, thereby guiding the selection of the final model (27).

Subgroup analyses were performed according to age, sex, and family history of diabetes. Multiplicative interactions were assessed by including interaction terms in the regression models. Among the 19,981 participants, smoking status had 15,214 missing values (76.14%), and drinking status had 15,214 missing values (76.14%). Because the missing rates for these two variables were extremely high, they were not included in the adjusted models to prevent potential adjustment bias. To evaluate the robustness of the findings to unmeasured confounding, sensitivity analyses calculated *E*-values to explore the potential impact of unmeasured confounding on the association between the CHG index and the risk of prediabetes and diabetes in normal-weight middle-aged and older Chinese adults (28). The *E*-value quantifies the magnitude of unmeasured confounding required to explain away this observed association.

## Results

### Characteristics of study participants

As shown in Table 1, this study enrolled 19,981 participants (mean age: 55.73 ± 9.04 years; 42.81% male), with a mean follow-up of 3.19 years. When the cohort was divided according to CHG index quartiles (Q1–Q4), those in the uppermost quartile (Q4) showed significantly elevated values for age, SBP, DBP, BMI, TC, FPG, LDL-C, ALT, BUN, and Scr in comparison to the lower quartiles (all *p* < 0.001), whereas HDL-C was substantially reduced (*p* < 0.001). The Q4 group also contained more men and active smokers (both *p* < 0.001), yet no inter-quartile differences emerged for drinking status or family history of diabetes (*p* = 0.101 and 0.079, respectively).

**Table 1:** The baseline characteristics of participants.

Variable	Total	CHG index quartile				*p*-value
		Q1 (3.98–4.85)	Q2 (4.85–5.01)	Q3(5.01–5.18)	Q4 (5.18–7.36)	
Participants	19,981	4,995	4,995	4,995	4,996	
Age (years)	55.73 ± 9.04	54.24 ± 8.68	55.07 ± 8.96	56.25 ± 9.20	57.36 ± 9.02	<0.001
SBP (mmHg)	120.15 ± 17.29	117.37 ± 16.70	119.02 ± 17.10	121.65 ± 17.53	122.57 ± 17.32	<0.001
DBP (mmHg)	74.65 ± 10.73	73.14 ± 10.59	74.07 ± 10.77	75.34 ± 10.89	76.06 ± 10.44	<0.001
BMI (kg/m^2^)	21.81 ± 1.40	21.42 ± 1.44	21.73 ± 1.40	21.94 ± 1.35	22.16 ± 1.29	<0.001
TC (mmol/L)	4.99 ± 0.90	4.53 ± 0.76	4.79 ± 0.76	5.08 ± 0.80	5.57 ± 0.94	<0.001
FPG (mmol/L)	4.82 ± 0.47	4.54 ± 0.48	4.79 ± 0.43	4.92 ± 0.41	5.03 ± 0.38	<0.001
HDL-C (mmol/L)	1.44 ± 0.32	1.68 ± 0.32	1.49 ± 0.26	1.38 ± 0.23	1.19 ± 0.23	<0.001
LDL-C (mmol/L)	2.90 ± 0.69	2.48 ± 0.53	2.75 ± 0.53	3.00 ± 0.58	3.38 ± 0.75	<0.001
ALT (U/L)	19.77 ± 21.62	18.55 ± 33.38	18.90 ± 12.79	19.69 ± 15.74	21.96 ± 18.41	<0.001
BUN (mmol/L)	4.81 ± 1.22	4.72 ± 1.21	4.82 ± 1.22	4.85 ± 1.29	4.85 ± 1.18	<0.001
Scr (mmol/L)	68.11 ± 15.43	65.07 ± 13.81	67.47 ± 14.81	69.83 ± 16.79	70.08 ± 15.62	<0.001
Gender						<0.001
Male	8,553 (42.81%)	1702 (34.07%)	2004 (40.12%)	2,269 (45.43%)	2,578 (51.60%)	
Female	11,428 (57.19%)	3,293 (65.93%)	2,991 (59.88%)	2,726 (54.57%)	2,418 (48.40%)	
Smoking status						<0.001
Current smoker	1,235 (25.91%)	228 (20.43%)	285 (24.87%)	302 (25.44%)	420 (31.87%)	
Ever smoker	127 (2.66%)	19 (1.70%)	26 (2.27%)	45 (3.79%)	37 (2.81%)	
Never smoker	3,405 (71.43%)	869 (77.87%)	835 (72.86%)	840 (70.77%)	861 (65.33%)	
Drinking status						0.101
Current drinker	142 (2.98%)	25 (2.24%)	37 (3.23%)	37 (3.12%)	43 (3.26%)	
Ever drinker	621 (13.03%)	121 (10.84%)	157 (13.70%)	154 (12.97%)	189 (14.34%)	
Never drinker	4,004 (83.99%)	970 (86.92%)	952 (83.07%)	996 (83.91%)	1,086 (82.40%)	
Family history of diabetes						0.079
No	19,549 (97.84%)	4,879 (97.68%)	4,874 (97.58%)	4,909 (98.28%)	4,887 (97.82%)	
Yes	432 (2.16%)	116 (2.32%)	121 (2.42%)	86 (1.72%)	109 (2.18%)	
Follow-up (years)	3.19 ± 0.95	3.24 ± 0.97	3.13 ± 0.94	3.16 ± 0.93	3.25 ± 0.96	<0.001

Results are presented as Mean ± SD for continuous variables and N (%) for categorical variables. CHG index, cholesterol, high-density lipoprotein, and glucose index; SBP, systolic blood pressure; DBP, diastolic blood pressure; BMI, body mass index; TC, total cholesterol; HDL-C, high-density lipoprotein cholesterol; LDL-C, low-density lipoprotein cholesterol; ALT, alanine aminotransferase; Scr, serum creatinine; BUN, blood urea nitrogen; FPG, fasting plasma glucose. Among the 19, 981 participants, SBP had 5(0.03%) missing values, DBP had 5(0.03%) missing values, LDL-C had 50(0.25%) missing values, ALT had 73(0.37%) missing values, BUN had 543(2.71%) missing values, Scr had 187(0.94%) missing values, smoking status had 15,214 (76.14%) missing values, and drinking status had 15,214 (76.14%) missing values.

### Tendency of prediabetes and diabetes across CHG index quartiles in normal-weight Chinese adults of middle and older age

Figure 2 presents the Kaplan–Meier survival curves for prediabetes and diabetes incidence stratified by CHG index quartiles among normal-weight Chinese adults of middle and older age. The four curves demonstrated evident divergence from the early follow-up period onward, indicating a significant link between elevated CHG index and increased risk of prediabetes and diabetes (log-rank test, *p* < 0.0001). Specifically, participants belonging to the lowest quartile (Q1) had the highest event-free survival probability across all time points, in contrast to the highest quartile (Q4), which displayed the smallest probability; this disparity intensified over time. The numbers at risk annotated beneath the figure illustrate a gradual reduction in the study population from approximately 5,000 at baseline to fewer than 300 by year 5, a temporal pattern consistent with the declining trajectory of the survival curves.

**Figure 2:**
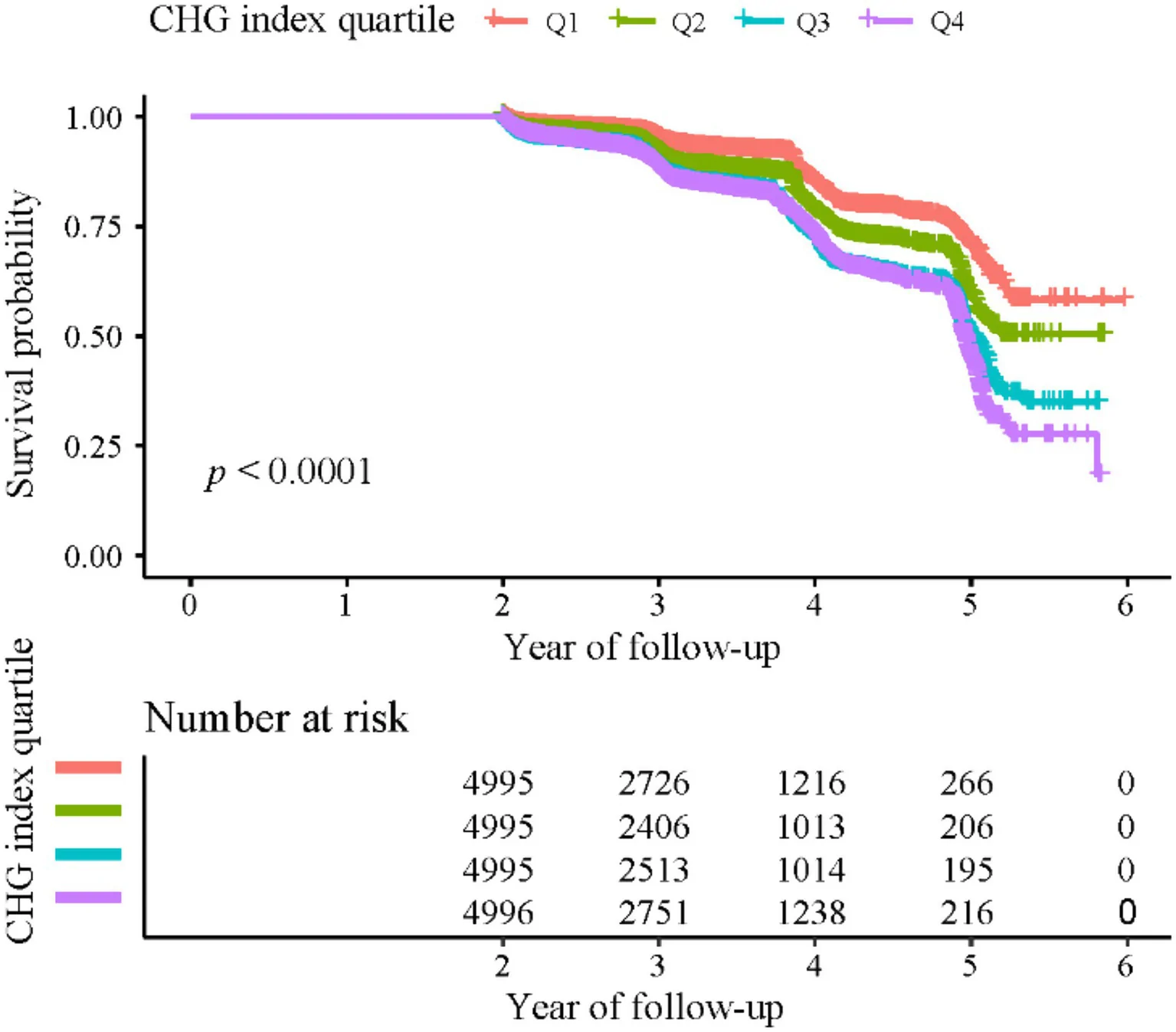
Kaplan–Meier eventfree survival curves for incident prediabetes and diabetes, stratified by CHG index quartiles, among- normalweight -middleaged a-nd older Chinese adults. The log-rank test revealed a significant difference across the quartile groups (*p* < 0.0001).

### Association between CHG index and incident prediabetes and diabetes in normal-weight Chinese adults of middle and older age

Cox proportional hazards models were employed to examine the relationship between the CHG index and incident prediabetes and diabetes among normal-weight Chinese adults of middle and older age (Table 2). In Model 1, a one-unit increment in the CHG index was linked to a markedly higher risk of the composite outcome (HR = 2.39, 95% CI: 2.13–2.68, *P* < 0.0001). Following adjustment for sex, SBP, age, and DBP (Model 2), the magnitude of this association diminished yet persisted with strong statistical significance (HR = 2.01, 95% CI: 1.78–2.26, *P* < 0.0001). Upon further controlling for family history of diabetes, BMI, LDL-C, Scr, BUN, and ALT in the fully adjusted Model 3, the CHG index continued to demonstrate a significant positive relationship with incident risk (HR = 2.20, 95% CI: 1.90–2.54, *P* < 0.0001). In quartile-based analyses with Q1 serving as the referent, individuals in Q2, Q3, and Q4 showed progressively higher risks, indicating a pronounced dose–response pattern; the corresponding HRs in Model 3 were 1.42 (95% CI: 1.26–1.60), 1.86 (95% CI: 1.65–2.08), and 1.91 (95% CI: 1.69–2.16), with P for trend remaining <0.0001 across all models.

**Table 2:** Association between CHG index and incident prediabetes and diabetes in normal-weight middle-aged and older Chinese adults.

Exposure	Model 1 (HR, 95% CI, *p*)	Model 2 (HR, 95% CI, *p*)	Model 3 (HR, 95% CI, *p*)
CHG index	2.39 (2.13, 2.68) < 0.0001	2.01 (1.78, 2.26) < 0.0001	2.20 (1.90, 2.54) < 0.0001
CHG index quartile			
Q1	Ref.	Ref.	Ref.
Q2	1.47 (1.31, 1.65) < 0.0001	1.40 (1.24, 1.56) < 0.0001	1.42 (1.26, 1.60) < 0.0001
Q3	2.02 (1.81, 2.24) < 0.0001	1.80 (1.61, 2.00) < 0.0001	1.86 (1.65, 2.08) < 0.0001
Q4	2.09 (1.88, 2.32) < 0.0001	1.81 (1.62, 2.01) < 0.0001	1.91 (1.69, 2.16) < 0.0001
*p* for trend	<0.0001	<0.0001	<0.0001

Model 1: unadjusted. Model 2: Model 1 + gender, age SBP and DBP. Model 3: Model 2 + family history of diabetes, BMI, LDL-C, Scr, BUN, and ALT; CHG index, cholesterol, high-density lipoprotein, and glucose index.

### Nonlinear relationship and threshold effect

Following multivariable adjustment (Model 3), smoothed curve fitting revealed a nonlinear association between the CHG index and the risk of prediabetes and diabetes among normal-weight middle-aged and older Chinese adults (Figure 3). A significant positive association emerged below the inflection point of 5.07 (HR = 6.39, 95% CI 4.73–8.64; *P* < 0.0001), whereas the curve flattened above this cutoff, with no discernible risk trend detected (HR = 1.02, 95% CI 0.79–1.31; *p* = 0.8740). The log-likelihood ratio test further corroborated this nonlinear relationship (*p* < 0.001) (Table 3).

**Figure 3:**
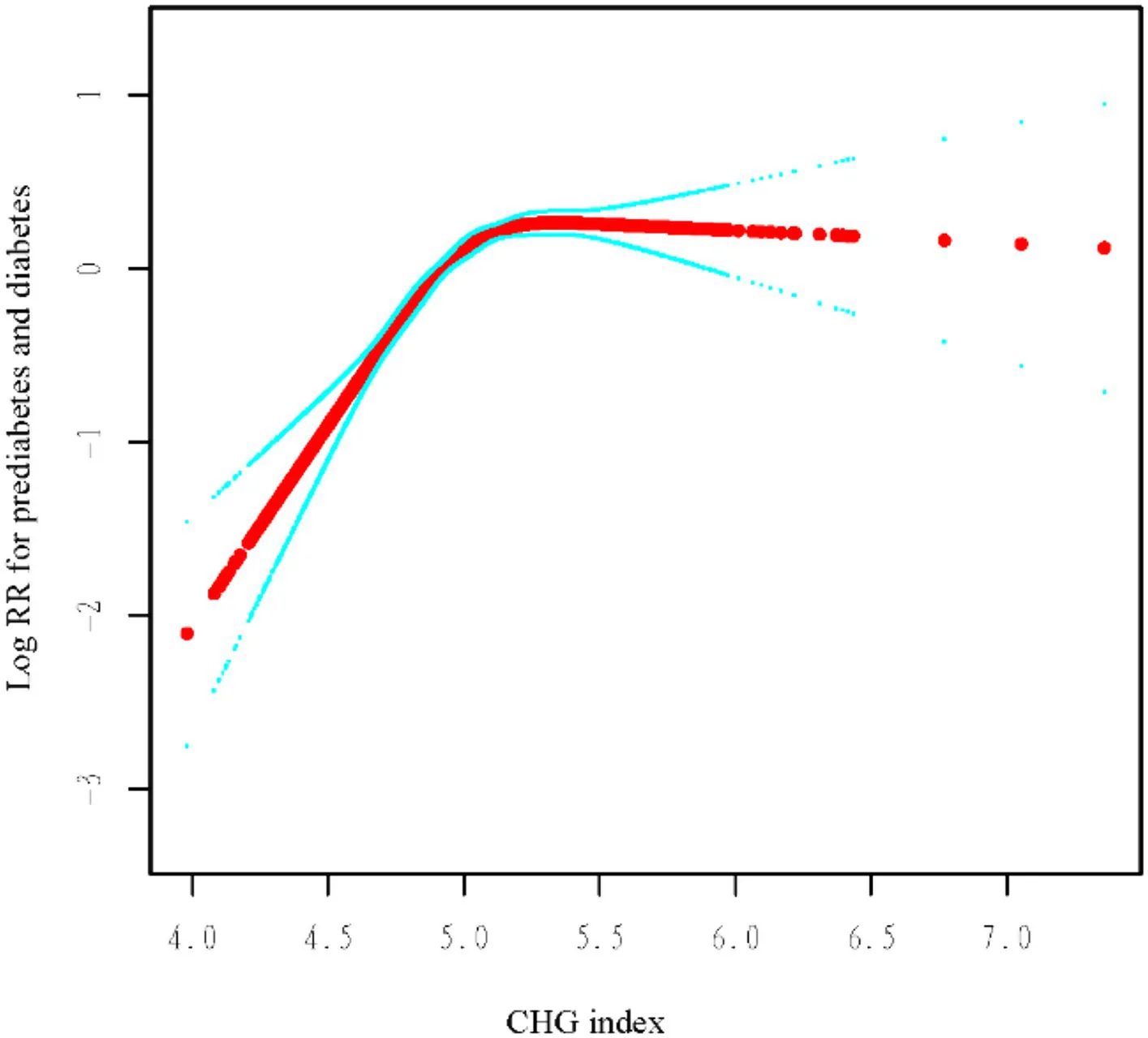
The nonlinear association between CHG index and incident prediabetes and diabetes in normal-weight middle-aged and older Chinese adults. A nonlinear relationship between them was detected after adjusting for gender, BMI, age, SBP, BUN, family history of diabetes, LDL-C, DBP, Scr, and ALT. The red curve on the graph represents the smoothed fit between the variables, while the blue lines indicate the 95% confidence intervals surrounding the fit.

**Table 3:** Threshold effect analysis between CHG index and incident prediabetes and diabetes in normal-weight middle-aged and older Chinese adults.

Threshold effect analysis	Outcomes
	HR (95%CI) *p*-value
Inflection point of CHG index (*K*)	5.07
<*K* slope	6.39 (4.73, 8.64) < 0.0001
>*K* slope	1.02 (0.79, 1.31) 0.8740
Log-likelihood ratio test	<0.001

The above model was adjusted for age, gender, SBP, DBP, family history of diabetes, BMI, LDL-C, Scr, BUN, and ALT; CHG index, cholesterol, high-density lipoprotein, and glucose index.

### Subgroup analyses

Stratified analyses (Table 4) revealed that the CHG index–outcome association was more pronounced among participants under 60 years of age (HR: 2.51, 95% CI: 2.10–3.00) than in those aged 60 years or above (HR: 1.65, 95% CI: 1.30–2.09), with a significant interaction (*p* = 0.0054). A similar pattern emerged by sex, where the association was stronger in women (HR: 2.65, 95% CI: 2.22–3.16) relative to men (HR: 1.77, 95% CI: 1.45–2.14) (P for interaction = 0.0011). By contrast, the association did not differ materially between participants with and without a family history of diabetes, as reflected by a non-significant interaction term (*p* = 0.5640).

**Table 4:** Subgroup analysis.

Characteristic	HR (95%CI)	*p* value	*p* interaction
Age			0.0054
45≥, <60	2.51 (2.10, 3.00)	<0.0001	
≥60	1.65 (1.30, 2.09)	<0.0001	
Gender			0.0011
Male	1.77 (1.45, 2.14)	<0.0001	
Female	2.65 (2.22, 3.16)	<0.0001	
Family history of diabetes			0.5640
No	2.19 (1.89, 2.53)	<0.0001	
Yes	2.85 (1.18, 6.84)	0.0195	

The model was adjusted for sex, age, SBP, DBP, family history of diabetes, BMI, LDL-C, Scr, BUN, and ALT. In subgroup analyses, the stratification variable was not included as a covariate in the model. CHG index, cholesterol, high-density lipoprotein, and glucose index.

### Sensitivity analyses

An *E*-value was calculated to assess sensitivity to unmeasured confounding. The derived *E*-value of 3.82 suggests that unmeasured or unknown confounders are unlikely to fully explain away the observed association between the CHG index and the risk of prediabetes and diabetes.

## Discussion

Our analysis of this large-scale, multicenter retrospective cohort of normal-weight, middle-aged and older Chinese adults revealed a significant non-linear positive association linking the CHG index to the risk of incident prediabetes and diabetes. Specifically, the association intensified steeply below a CHG value of 5.07 and flattened above this point, with this pattern persisting after controlling for numerous covariates.

Our findings are in broad agreement with earlier studies that have established a positive link between the CHG index and dysglycemia. Mansoori et al. originally proposed the CHG index as a diagnostic surrogate for type 2 diabetes, and subsequent cross-sectional and longitudinal investigations have confirmed its predictive value for prevalent and incident diabetes (23, 29, 30). However, our study differs from previous CHG research by focusing on normal-weight (BMI 18.5–23.9 kg/m^2^) middle-aged and older adults—a population largely overlooked despite its substantial metabolic vulnerability. Most prior studies included participants across all BMI categories or focused on overweight/obese individuals (18, 31). We observed that the association was stronger in females and in younger participants (<60 years), patterns that have not been consistently reported in previous CHG studies. Several reasons may account for these differences. In normal-weight individuals, traditional anthropometric indicators such as BMI are less informative; therefore, metabolic signals captured by the CHG index—particularly the interplay between lipids and glucose—may become more prominent in identifying high-risk individuals (32, 33). The stronger association in women may reflect sex differences in body fat distribution, which are well documented and linked to differential cardiometabolic risk (34). The age-related attenuation (weaker association in those ≥60 years) might reflect competing age-related changes, such as sarcopenia, hormonal shifts, and cumulative exposure to multiple risk factors, which could dilute the relative contribution of the CHG index in older age (35). In older adults, mitochondrial dysfunction and altered substrate metabolism may further obscure the relationship between circulating glycolipid markers and incident dysglycemia (36–38).

The observed relationship between the CHG index and incident prediabetes and diabetes can be mechanistically linked to the core components of the index—TC, HDL-C, and FPG—which collectively mirror several interconnected pathophysiological processes (23). First, elevated fasting glucose directly reflects impaired hepatic glucose homeostasis and progressive β-cell dysfunction, both of which are central to the development of diabetes (39, 40). Second, the combination of raised TC and reduced HDL-C is a hallmark of the atherogenic dyslipidemia that frequently accompanies insulin resistance (29, 41). In insulin-resistant states, increased hepatic *de novo* lipogenesis and very-low-density lipoprotein secretion, along with reduced reverse cholesterol transport, lead to an unfavorable lipid profile (42). These lipid abnormalities not only promote atherosclerosis but also exacerbate pancreatic lipotoxicity, further compromising β-cell function (43). The multiplicative integration of these parameters in the CHG index may capture a more holistic metabolic disturbance than any single marker. The threshold effect (steep rise below CHG = 5.07 and plateau above) suggests a biological saturation phenomenon. Below the threshold, incremental increases in the CHG index—driven by worsening glycaemia, rising cholesterol, and falling HDL-C—likely reflect accelerating insulin resistance and progressive β-cell exhaustion, leading to a disproportionately rapid increase in disease risk (3, 17). Once the metabolic dysregulation surpasses a critical severity (i.e., beyond the inflection point), the system may have already entered a state of advanced decompensation, where additional increments in the index no longer translate into commensurate risk elevation, possibly due to biological saturation or competing physiological feedback mechanisms (14). Inflammatory pathways and oxidative stress, which are intimately linked to both dyslipidaemia and hyperglycaemia, may also play a mediating role, as chronic low-grade inflammation is known to promote insulin resistance and β-cell apoptosis (44). Beyond the lipid-glucose axis, accumulating evidence implicates Sirtuin family proteins in the pathogenesis of diabetic complications. SIRT3 deficiency has been shown to exacerbate diabetic cardiomyopathy through enhanced necroptosis and NLRP3 inflammasome activation (45), while Sirt1 activation attenuates oxidative stress and inflammation in retinal Müller cells under hyperglycemic conditions (46). Andrographolide, a diterpenoid lactone with anti-inflammatory properties, has demonstrated neuroprotective effects in experimental models (47), and dendrobium polysaccharide-based biomaterials have shown promise in promoting wound healing while reducing inflammation (48). In addition, sulforaphane, a naturally occurring isothiocyanate, exerts antioxidant and anti-inflammatory effects via Nrf2 activation and has been reported to reverse diabetes-induced cognitive and memory deficits (49). Collectively, these findings suggest that the metabolic dysregulation captured by the CHG index may propagate end-organ damage through oxidative stress, inflammatory cascades, and programmed cell death pathways, and that targeting these mechanisms may offer adjunctive therapeutic avenues for this population.

Our study presents several notable strengths. The use of data from a large-scale, multi-center cohort in China underpins the robustness and generalizability of our evidence. To the best of our knowledge, this represents the first attempt to evaluate the CHG index–glucose dysregulation association specifically in non-obese, middle-aged and older Chinese adults. Moreover, the observed non-linear positive relationship between the CHG index and incident prediabetes/diabetes offers new information on the dose–response nature and possible thresholds, which could inform future approaches to risk assessment and targeted prevention.

We also acknowledge several limitations. First, a limitation of this retrospective observational cohort study is that causality regarding the CHG index and its relationship with prediabetes/diabetes risk cannot be established; the findings should therefore be interpreted as associations rather than causal effects. Second, because the study population was exclusively Chinese, the results may not be generalizable to other ethnic groups or geographic regions. Third, despite extensive covariate adjustment, residual confounding from unmeasured factors—such as diet, physical activity, and socioeconomic status—may have influenced the results. In addition, because missing data for smoking and drinking status were substantial (>76%), these variables were not included in the adjusted models, potentially introducing bias. As with any observational study, the potential for residual confounding stemming from unmeasured factors cannot be entirely ruled out. Nevertheless, using *E*-values, we estimated that unmeasured confounding was unlikely to substantially affect our findings. Finally, the mean follow-up of 3.19 years may not capture long-term risk trajectories. Therefore, limitations related to study design, population, and potential confounding should be addressed in future research.

## Conclusion

Our analysis of this large, multicenter retrospective cohort, which focused on normal-weight Chinese adults of middle and older age, revealed a significant non-linear positive relationship linking the CHG index to the incidence of prediabetes and diabetes. This simple, low-cost metabolic indicator derived from routine laboratory tests may facilitate the identification of metabolically vulnerable individuals within this traditionally overlooked population, supporting earlier risk stratification strategies.

## Data Availability

Publicly available datasets were analyzed in this study. This data can be found here: https://datadryad.org/stash/dataset/doi:10.5061%2Fdryad.ft8750v.

## Footnotes

1
https://datadryad.org/stash/dataset/doi:10.5061/dryad.ft8750v
